# Safety and effectiveness of oral propranolol for infantile hemangiomas started before 5 weeks and after 5 months of age: an Italian multicenter experience

**DOI:** 10.1186/s13052-017-0357-9

**Published:** 2017-04-19

**Authors:** Maya El Hachem, Francesco Gesualdo, Andrea Diociaiuti, Irene Berti, Nadia Vercellino, Valeria Boccaletti, Iria Neri, Giulio Porcedda, Antonella Greco, Claudia Carnevale, Teresa Oranges, Mario Cutrone, Pietro Dalmonte

**Affiliations:** 1 Pediatric Dermatology Unit, Bambino Gesù Children’s Hospital, IRCCS, Rome, Italy; 2 Multifactorial and Complex Disease Research Area, Bambino Gesù Children’s Hospital IRCCS, Viale Baldelli 41, 00146 Rome, Italy; 3 Institute for Maternal and Child Health - IRCCS Burlo Garofolo, Trieste, Italy; 4 Angioma Center, Cardiovascular Department, Gaslini Children’s Research Institute, Genoa, Italy; 5 Dermatology Clinic, Parma University Hospital, Parma, Italy; 6 Division of Dermatology, Department of of Experimental, Diagnostic and Specialty Medicine, University of Bologna, Bologna, Italy; 7 Pediatric Cardiology Unit, Anna Meyer Children’s Hospital, Florence, Italy; 8 Pediatric Emergency Department, Ospedale dell’Angelo Ulss12, Venice, Italy

## Abstract

**Background:**

Despite not being licensed for the treatment of infantile hemangiomas (IH) in infants younger than 5 weeks or older than 5 months, propranolol is often used in these age groups to prevent or to treat potentially severe complications. The objective of the present study was to review the experience of 8 Italian pediatric and dermatologic centers regarding propranolol treatment for IH started before 5 weeks or after 5 months of age.

**Methods:**

We retrospectively reviewed the records of patients followed up for IH, on propranolol treatment started before 5 weeks or after 5 months of age, and collected information on sociodemographic data, treatment indications, IH involution, IH relapse, and treatment side effects.

**Results:**

A total of 343 patients were enrolled; 15 were started on propranolol before 5 weeks (group 1), 328 were started after 5 months of age (group 2). The most frequent indications were permanent aesthetical disfigurement (91.8%) and function threatening complications (42.6%).

In most cases, the treatment was effective. The involution was partial in 67.7% of patients. In 11.8% of cases a relapse was observed. No relapse was observed in group 1. Treatment complications were reported in 15.8% of children, most frequently sleep disorders (6.6%), followed by irritability (5.1%) and diarrhea (2.2%). Only a case of mild constipation was observed in group 1.

**Conclusion:**

The safety and effectiveness profile of propranolol in infants younger than 5 weeks or older than 5 months may be acceptable. Taking in account propranolol's potential in preventing severe complications, further studies should assess the acceptability of propranolol treatment, especially in the <5-week age group .

## Background

Infantile hemangiomas (IHs) are the most common benign neoplasms in infancy [1–5]. The natural history of IHs is characterized by an onset within the first weeks of life, followed by a proliferative phase with rapid growth, generally lasting until 5 months of age; in some cases, the proliferative phase may last up to 10-11 months [6].

About 90% of IHs spontaneously regress, partially or completely, within the 7th year of life (usually between the 3rd and the 6th year) [2, 7, 8]. In 10-15% of cases, a treatment is required, with the following indications: life threatening complications, function threatening complications, ulceration not responding to local treatments, pain and potential permanent disfigurement [6, 9–15].

Oral propranolol has been repeatedly demonstrated as the first-choice drug for IH and, at present, is available in several countries with a specific indication for pediatric patients [6, 16, 17].

The treatment is highly effective (up to 96-98% after 6 months) and has an optimal safety profile, with a low incidence of adverse events, such as sleep disorders, diarrhea, cold extremities, irritability, hypotension, bradycardia, bronchospasm and hypoglycemia. Symptomatic hypotension and bradycardia are very rare [17–19]. All side effects are usually transient and only in few cases a definitive treatment discontinuation is required [20].

According to international consensus recommendations and to the drug’s summary of product characteristics (SmPC), oral propranolol should be administered in infants between 5 weeks and 5 months of age, for a duration shorter than 6 months [6]. Nevertheless, peculiar clinical pictures may exist, in which an off-label treatment could be required or suggested.

The aim of our study was to describe the experience of 8 Italian pediatric and dermatologic centers in the off-label use of oral propranolol for the treatment of IH, starting earlier than 5 weeks or later than 5 months of age, in terms of treatment indications, effectiveness and side effects.

## Methods

### Study design and population

This is a multicenter, retrospective cohort study that was conducted in the Pediatric Units and Pediatric Dermatology Units of 8 major Italian metropolitan hospitals.

We retrospectively reviewed the records of patients followed up for IH in the participating centers between June, 2008 and June, 2015, who had been treated with oral propranolol started before 5 weeks or after 5 months of age, and who had discontinued the treatment for at least one month. The following data were collected: sociodemographic data; risk factors for IH; IH therapy (indication, dose, therapy duration); treatment effectiveness (involution at treatment end, relapse); treatment complications (including irritability, nightmares, cold extremities, diarrhea, hypoglycemia, hypotension).

Data were collected in a dedicated database.

The study was approved by the Ethical Committee of the Bambino Gesù Children’s Hospital.

### Statistical analysis

Data were described as means and SD or medians and range or proportions and CI, as appropriate. Age is expressed in months, and was corrected to 40 weeks of gestational age for preterm infants.

Stata 13 was used for the statistical analysis.

## Results

### Study population

We enrolled a total of 343 patients. Characteristics of the study population are presented in Table 1.

**Table 1:** Sociodemographic characteristics and IH risk factors

	Start <5 weeks (*N* = 15)	Start >5 months (*N* = 328)	Total (*N* = 343)
Female sex (N, %)	10 (66.7)	232 (71)	242 (70.8)
Age at treatment start (median, range)	0.8 (0-1.1)	9 (5-90.7)	8.7 (0-90.7)
Age at treatment end (median, range)	10 (7.3-15.2)	16.1 (8.6-107.1)	16 (7.3-107.1)
Treatment duration (median, range)	9.5 (6.3-14.5)	6.8 (0.5-20)	6.8 (0.5-20)
Preterm (N, %)	8 (53.3)	49 (14.9)	57 (16.6)
Risk factors (N, %)	9 (60)	73 (22.3)	82 (24)
twins	3 (20)	16 (4.8)	19 (5.5)
IVF	1 (6.7)	2 (0.6)	3 (0.9)
family history of IE	0 (0)	9 (2.7)	9 (2.6)
amniocentesis	1 (6.7)	15 (4.6)	16 (4.7)
villocentesis	2 (13.3)	10 (3)	12 (3.5)
pre-eclampsia or placenta praevia	2 (13.3)	12 (3.7)	14 (4)

A total of 242 (70.8%) were female.

Fifteen patients (4.3%) were started on oral propranolol before 5 weeks of age; 328 patients (96%) had started the treatment after 5 months of age.

Median age at treatment start was 0.8 months in the subgroup of patients starting the therapy before 5 weeks of age (range 0-1.1 months) and 9 months in the subgroup of patients starting the therapy after 5 months (range 5-90.7 months).

Median treatment duration was 9.5 months (range 6.3-14.5 months) among patients starting the therapy before 5 weeks of age and 6.8 months (range 0.5-20 months) in those starting the therapy after 5 months. A patient was treated with propranolol for 2 weeks only, waiting to be treated surgically. Despite the short interval between the treatment start and the surgical intervention, the clinicians decided to treat the patient anyway, in order to reduce the IH's vascularization and the consequent risk for an intraoperative hemorrhage.

A total of 57 children (16.6%) were born preterm. The proportion of preterms among patients starting the treatment before 5 weeks of age was high (53%).

At least one risk factor among those recognized in the medical literature [6, 21] and listed in Table 1 was identified in 82 patients (24%), most commonly twin pregnancy (5.5%), amniocentesis (4.7%) and pre-eclampsia or placenta previa (4%). Risk factors were reported in a high proportion (60%) of patients starting the treatment before 5 weeks and in 22.3% of patients starting the treatment after 5 months. Twin pregnancy was the most common risk factor in both groups.

### Treatment indications and dosages

Treatment indications are reported in Table 2.

**Table 2:** Treatment indications

	Start <5 weeks (*N* = 15)	Start >5 months (*N* = 328)	Total (*N* = 343)
Life threatening complications	2 (13)	4 (1.2)	6 (1.8)
Function threatening complications	5 (66.7)	136 (41.4)	146 (42.6)
Permanent esthetical disfigurement	13 (86.7)	302 (92.1)	315 (91.8)
Ulceration	3 (20)	8 (2.4)	11 (3,2)
Pain	2 (13.3)	4 (1.2)	6 (1.8)

In both groups, the most frequent indications to treatment with propranolol was potential permanent aesthetical disfigurement, followed by function threatening complications. Other indications accounted for less than 5% in the subgroup started on propranolol after 5 months of age. On the other hand, among infants starting the treatment before 5 weeks, life threatening, ulceration and pain were reported in a total of 5 patients (1/3 of the subgroup). Of the subgroup of 6 patients with a life threatening indication, the following localizations were reported: laryngeal (3 patients), liver (3 patients), and hemithorax (1 patient). All patients started on propranolol before 5 weeks of age for life threatening complications had a liver hemangiomatosis.

Median therapeutic dosage was 2 mg/Kg daily (range 1-3). A proportion of 54% patients had a dosage between 1.8 and 2.2 mg/Kg. As per protocol, children <5 weeks were started on a lower dosage and progressively adjusted.

### Effectiveness and safety

In most cases, the treatment was effective, although the involution at the end of the treatment was partial in 2/3 of patients, in both groups (Table 3).

**Table 3:** Effectiveness and safety

	Start <5 weeks (*N* = 15)	Start >5 months (*N* = 328)	Total (*N* = 343)
Involution			
complete	2 (13.3)	77 (23.9)	79 (23.4)
almost complete	3 (20)	17 (5.3)	20 (5.9)
partial	10 (66.7)	218 (67.7)	228 (67.7)
absent	0	10 (3.1)	10 (3)
Relapse	1 (6.7)	39 (12)	40 (11.8)
Treatment complications	1 (6.7)	52 (15.9)	53 (15.5)
irritability	0	17 (5.4)	17 (5.12)
sleep disorders	0	22 (6.8)	22 (6.6)
cold extremities	0	2 (0.6)	2 (0.6)
diarrhoea	0	10 (3.1)	10 (2.9)
hypoglicemia	0	1 (0.3)	1 (0.3)
hypotension	0	4 (1.3)	4 (1.2)

Among the 15 patients started on propranolol before 5 weeks, an involution was seen in all patients (complete: 2 patients; almost complete: 3 patients; partial: 10 patients).

Among patients started on propranolol after 5 months, a complete or almost complete involution at treatment end was observed in 23.9% and 5.3%, respectively. Only in 3% of cases no involution was observed.

Relapse was infrequent among patients started on treatment before 5 weeks (recorded in 1 patient only). Twelve patients in the older group had a relapse.

Treatment complications were reported in 15.8% of children, mostly children started on propranolol after 5 months of age.

In this group, the most frequent complications were sleep disorders (6.8%), followed by irritability (5.4%) and diarrhea (3.1%). Cold extremities, hypoglycemia, hypotension and wheezing were observed in a very low proportion of cases. All the described cases of hypoglicemia and hypotension were asymptomatic. A severe complication was reported in a child started on propranolol at 12 months of age for a wide IH of the back, with a high risk of ulceration. The patient had a cardiac arrest after 40 days of treatment, during a respiratory tract infection.

Among patients starting the treatment before 5 weeks of age, only a case of mild constipation was observed.

## Discussion

With the present descriptive study, we show that the off-label use of oral propranolol for IHs in infants, with treatment start before 5 weeks or after 5 months of age, may have an acceptable profile of effectiveness and safety.

Based on the only randomized controlled trial (RCT) available in the literature [19], the indications for oral propranolol in infants suggest a treatment start between 5 weeks and 5 months.

Off-label use of propranolol after 5 months of age is frequent and already described in the literature [22].

As a matter of fact, despite children with an IH requiring treatment should be started on oral propranolol within the first 2-3 months of age, referral to dermatology centers may be late (after 5 months), due to a scarce knowledge of the therapeutic option of propranolol, and of its safety and efficacy, among general pediatricians. Moreover, despite the regression of IH is usually spontaneous after 1 year of age, sometimes complications may be severe and waiting for a spontaneous involution may cause irreversible functional and aesthetical damage. In infants referred to pediatric dermatology centers after 5 months of age, propranolol, through its pro-apoptotic properties, may promote and accelerate the involution of the lesion, thus reducing the risk of permanent damages.

On the other hand, a treatment start before 5 weeks of age is less frequently reported. Although very rarely, IHs may actually pose a risk of severe and possibly irreversible complications at birth or within the first 5 weeks of life, thus justifying an early treatment start, with an indication of life or function threatening complications, eg. laryngeal hemangiomas impairing the breathing dynamic, periorbital hemangiomas entailing a risk of amblyopia and liver hemangiomas impairing the hemodynamics, with a high risk of cardiac failure. When starting the treatment before 5 weeks, special precautions should be carefully taken, accordingly to consensus guidelines [6, 23]: hospitalization at treatment start and during dose adjustment, low initial dose, continuous monitoring of blood pressure and heart rate for the first week, follow up visits every 2-4 weeks.

The experience of the major Italian pediatric and dermatologic centers reported in the present article confirms an acceptable profile of the drug in terms of effectiveness, safety and prevention of IH-related complications in children younger than 5 weeks and older than 5 months.

Effectiveness was very high. Any improvement (including partial, almost complete and complete involution) was reported in 97% of the study population. The authors of a systematic review published in 2013 and including 41 articles on propranolol treatment for IH reported a mean response rate of 98% (ranging from 82% to 100%), which is consistent with our figures. Nevertheless, according to this review, rebound growth and adverse events were more frequent compared to our population (rebound growth = 17% compared to 11.8% in our population; adverse events = 30% compared to 15.8% in our population) [24].

In a recent article on propranolol safety, the reported complication rate was lower (8.8%) compared to our figures [25]. The reason of this lower proportion of treatment complications is probably due to the fact that the authors assessed the causality using a standardized protocol [26].

In our population, 15 children were started on propranolol before 5 weeks of age, mainly due to life-threatening complications, ulceration and pain. In this group, risk factors were highly represented, especially preterm birth and twin pregnancy. Efficacy was high, as involution of the lesion was observed in all cases started on treatment before 5 weeks, and only one case of relapse was described, in a child treated for a parotideal IH. Parotideal IHs are known to exhibit an extended growth phase during the second and third year of life. This could explain the frequent relapse at treatment discontinuation [19, 27]. As a matter of fact, the low proportion of relapse in this subgroup may be related to the fact that the treatment duration was always longer than 6 months (median 9.5 months). The SmPC suggests a treatment duration shorter than 6 months. However, our data suggest that the safety and effectiveness of a longer treatment duration in terms of reduction of relapse may be convenient and should be further investigated. Moreover, no side effect of the treatment was observed in this subgroup, apart from a mild case of constipation. We believe that the extreme accuracy of the management of these very young infants, with slow dosage increase and continuous monitoring of vital signs, has strongly contributed to the prevention of side effects.

Among the subgroup of patients started on propranolol after 5 months of age, treatment complications were reported in 16% of cases, mainly including sleep disorders and irritability. One severe adverse event was reported: a cardiac arrest during a respiratory tract infection in a child that had been treated for 40 days. The child suffered from breath-holding spells and was dehydrated due to the infection. Cardiac adverse events potentially related to propranolol therapy have been previously reported in the literature [25], and one case of cardiac arrest in an infant on propranolol with bronchiolitis has been described [28]. Based on these observations, parents should be carefully trained to recognize symptoms of a respiratory infection and advised to immediately discontinue treatment if a respiratory infection occurs, as previously reported [23].

The present study has several limitations, mainly with regards to its design. First of all, we did not compare our results with those obtained from a retrospective cohort of controls started on propranolol between 5 weeks and 5 months of age. Nevertheless, our results were in line with the figures reported in the literature. Moreover, causality of side effects has not been investigated with a standardized protocol, and this may have impaired our estimates, probably through an overestimation of the frequency of side effects. Finally, the subgroup of infants started on propranolol before 5 weeks of age was relatively small, and this may have affected the precision and generalizability of our estimates.

## Conclusion

In conclusion, we report an acceptable profile of effectiveness and safety for the off-label use of propranolol in infants, with figures that are comparable to those reported in the literature, with regard to involution, relapse and complications. In particular, relapse and complication rates in children started on propranolol at an age younger than 5 weeks were even lower than those reported for other age groups. Despite this subgroup of patient was relatively small, this finding suggests that a closely monitored therapy start may contribute to a higher safety profile in these very young infants. On the other hand, the late start of propranolol after 5 months of age is often due to the late referral of patients by family pediatricians, which are still not aware of the availability of propranolol therapy for IH. With the aim of resolving this cultural gap, in Italy, the Italian Society for the Study of Vascular Anomalies (SISAV) and the Italian Society of Pediatric Dermatology (SIDerP) promoted educational courses dedicated to pediatricians and dermatologists for the management of IHs.

Finally, based on our observations, additional RCTs are needed to validate the use of propranolol, especially in the <5-week age group, in order to reduce IH-related complications, which may be severe and irreversible.
